# Increased Internet Searches for Insomnia as an Indicator of Global Mental Health During the COVID-19 Pandemic: Multinational Longitudinal Study

**DOI:** 10.2196/22181

**Published:** 2020-09-21

**Authors:** Yu-Hsuan Lin, Ting-Wei Chiang, Yu-Lun Lin

**Affiliations:** 1 Institute of Population Health Sciences National Health Research Institutes Miaoli County Taiwan; 2 Institute of Health Behaviors and Community Sciences College of Public Health National Taiwan University Taipei Taiwan; 3 Department of Psychiatry National Taiwan University Hospital Taipei Taiwan; 4 Department of Psychiatry, College of Medicine National Taiwan University Taipei Taiwan

**Keywords:** internet search, Google Trends, infodemiology, infoveillance, COVID-19, insomnia, mental health

## Abstract

**Background:**

Real-time global mental health surveillance is urgently needed for tracking the long-term impact of the COVID-19 pandemic.

**Objective:**

This study aimed to use Google Trends data to investigate the impact of the pandemic on global mental health by analyzing three keywords indicative of mental distress: “insomnia,” “depression,” and “suicide.”

**Methods:**

We examined increases in search queries for 19 countries. Significant increases were defined as the actual daily search value (from March 20 to April 19, 2020) being higher than the 95% CIs of the forecast from the 3-month baseline via ARIMA (autoregressive integrated moving average) modeling. We examined the correlation between increases in COVID-19–related deaths and the number of days with significant increases in search volumes for insomnia, depression, and suicide across multiple nations.

**Results:**

The countries with the greatest increases in searches for insomnia were Iran, Spain, the United States, and Italy; these countries exhibited a significant increase in insomnia searches on more than 10 of the 31 days observed. The number of COVID-19–related deaths was positively correlated to the number of days with an increase in searches for insomnia in the 19 countries (ρ=0.64, *P*=.003). By contrast, there was no significant correlation between the number of deaths and increases in searches for depression (ρ=–0.12, *P*=.63) or suicide (ρ=–0.07, *P*=.79).

**Conclusions:**

Our analysis suggests that insomnia could be a part of routine mental health screening during the COVID-19 pandemic.

## Introduction

The COVID-19 pandemic is the largest global public health challenge of this century. The number of confirmed COVID-19 cases and deaths have been growing exponentially, with over 355,688 deaths and 5,695,115 people infected worldwide, as of May 28, 2020 [1]. In mid-March, countries with the highest prevalence of COVID-19 cases imposed lockdown policies, such as social distancing, bans on nonessential travel, and the temporary closure of almost all businesses, facilities, and places of religious and other gathering, including funerals. There are concerns that the pandemic and the secondary consequences of the public health response, such as lockdowns and social distancing, may adversely affect mental health. In addition, in the absence of a vaccine or effective treatment, fear of COVID-19 and increased experience of bereavement have left more people vulnerable to mental health problems, such as insomnia, anxiety, depression, posttraumatic stress, and suicide. The World Health Organization (WHO) has recognized the importance of considering population-level psychological well-being and mental health during the COVID-19 pandemic [2].

Surveys on COVID-19–related mental health have been mostly based on self-reported, cross-sectional studies. Most of these surveys investigated a single country; multinational studies have been scarce [3]. Only one study, with a repeated cross-sectional design, has monitored changes in a population’s mental health throughout the course of the pandemic [4], but there was no longitudinal follow-up survey. Therefore, current research still does not provide a strong basis for national mental health strategies. However, mental distress can be reflected in Google searches. For example, Google searches for flu symptoms have been found to be real-time indicators of influenza outbreaks [5]. Google Trends has been used previously for population mental health surveillance, and for longitudinal tracking to identify potential risk factors of depression [6] and suicide [7]. In a global crisis like the COVID-19 pandemic, real-time global mental health surveillance is urgently needed for tracking the long-term impact.

This study hypothesized that increases in search terms related to mental health might correspond to the prevalence of COVID-19 in different countries. We aimed to use Google Trends data to investigate the impact of the pandemic on populations’ mental health, as indexed by changes in search frequency for three keywords indicative of mental distress: insomnia, depression, and suicide. We delineate a 4-month time course and focus on the period from March 20 to April 19, 2020, to evaluate search behaviors related to insomnia, depression, and suicide in response to a nation’s level of COVID-19 transmission.

## Methods

### Study Design

Using Google Trends data, we obtained search trends including “insomnia,” “depression,” and “suicide” from December 20, 2019, to April 19, 2020, as a surrogate for the general population’s mental health status in 19 countries with differing rates of COVID-19 prevalence. We confirmed the translation of the three terms into the local languages of the 19 countries by using translations from both Chinese and English, with back-translation on Google Translate.

Google Trends does not provide information on the absolute numbers of searches. Instead it provides a relative search value to display search activity for a given term according to a specific period, time, and area. Each data point is divided by the total searches of the geography and time range it represents to compare relative popularity. This value is scaled from 0 to 100. A value of 100 is the peak popularity of the term, while a value of 50 means that the term is half as popular in a given time period/area with search volumes for the days given relative to this.

We examined daily searches from March 20 to April 19, using this period since most countries enforced lockdowns in mid-March, to compare observed search volumes with expected search volumes from the 3-month baseline. The 3-month baseline period from December 20, 2019, to March 20, 2020, included two sentinel events: (1) on January 20, Chinese health authorities announced the human-to-human transmission of COVID-19, (ie, the first baseline month prior to any widespread knowledge of the disease worldwide); and (2) on February 20, the number of cases of COVID-19 outside China started to increase rapidly (no countries other than China had accumulated more than 100 cases of COVID-19 prior to February 19). The 3-month baseline time window was selected to inform our prediction since a longer time window could be contaminated by other past relevant events.

### Increased Search Volume Estimation

Using search rates from December 20, 2019, to March 19, 2020, for each outcome (“insomnia,” “depression,” or “suicide”), we forecasted a counterfactual scenario of expected search rates had the COVID-19 rapid outbreak and lockdown policies not occurred in mid-March. The expected relative search volumes were estimated using Hyndman and Khandakar’s algorithm for autoregressive integrated moving average (ARIMA) modeling [8], fit to the historical search rates from December 20, 2019, to March 19, 2020, and compared to the observed search rates from March 20 to April 19, 2020. For the residuals from each of the chosen models, we verified that they showed no significant autocorrelation with a Ljung–Box test [9]. Subsequently, we used daily trends from December 20, 2019, to March 19, 2020, to forecast future values with bootstrap CIs, which were computed using R software, version 3.6.3 (R Foundation for Statistical Computing).

### Indicators of Increased Searches for Insomnia, Depression, and Suicide

We examined the number of days with increased search volume for insomnia, depression, and suicide. We defined increases in search queries based on both the intensity and duration of increases in population interest, which was applied per our previous study [10]. The intensity of a significant increase was defined as the actual daily Google Trends value during March 20 to April 19 being higher than the expected +1.645 standard error (via ARIMA), that is, the upper limit of the 95% CI in a one-tailed test. We calculated the number of days with significant increases in searches for insomnia higher than the 95% CI expected in each country from March 20 to April 19. We used similar approaches to calculate the indicators for depression and suicide.

### Rate of COVID-19 Spread Across Nations

We used the increase in the number of deaths from March 20 to April 19 as an indicator of the speed at which COVID-19 spread within a nation during this time period, since widespread population testing was not yet available in most countries, especially in those with rapid outbreaks of COVID-19. However, in order to examine the robustness of our findings, we also used three additional indicators: increases in confirmed cases of COVID-19 from March 20 to April 19; the cumulative number of confirmed cases; and the cumulative number of deaths as of March 20.

We used the Spearman rank-order correlation test to examine the correlation between increases in the number of confirmed cases and deaths related to COVID-19 and the number of days with increases in search volume for insomnia, depression, and suicide in the 19 countries from March 20 to April 19. We also examined the temporal correlation between confirmed cases and deaths as of March 20 as well as the number of days with increases in search volumes for insomnia, depression, and suicide from March 20 to April 19.

## Results

**Figure 1:**
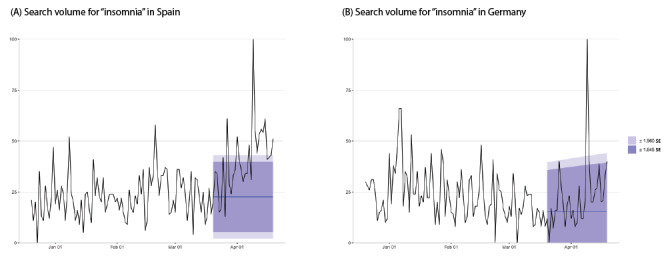
Daily trends for all Google searches for the term “insomnia” alongside expected trends for the days after March 20, 2020, in (A) Spain and (B) Germany.

**Figure 2:**
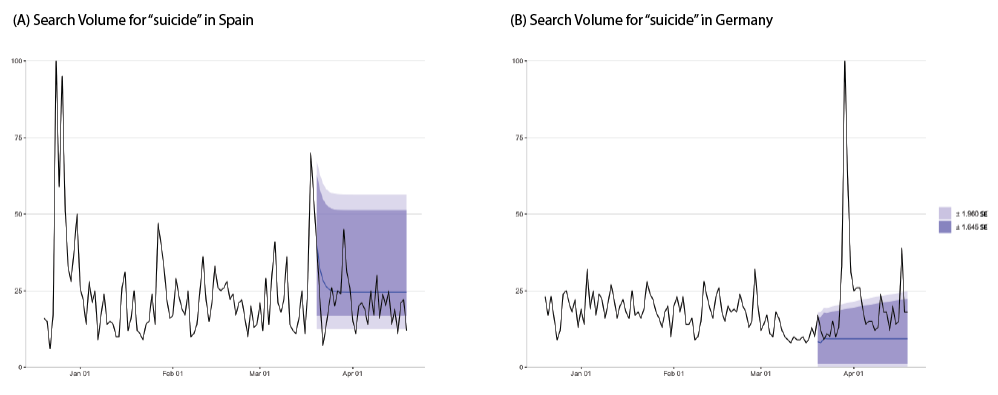
Daily trends for all Google searches with the term “suicide,” alongside expected trends for the days after March 20, 2020, in (A) Spain and (B) Germany.

Table 1 shows the number of days with significant increases in searches for insomnia, depression, and suicide in the local language among the 19 countries. Nations with the highest increases in searches for insomnia were Iran, Spain (Figure 1), the United States, and Italy; these countries exhibited a significant increase in insomnia queries on more than 10 of the 31 days observed (March 20 to April 19). The countries with the greatest increases in searches for depression were Iran (8 days), Australia (6 days), and Hong Kong (6 days). The countries with the greatest increases in searches for suicide were Iran (10 days), Germany (10 days [Figure 2]), and Italy (5 days).

**Table 1:** Number of days with significant increases in searches for insomnia, depression, and suicide in each country’s local language, as well as the cumulative number of confirmed cases as of March 20, 2020; increases in confirmed cases from March 20 to April 19; and the increases in the number of deaths due to COVID-19.

Country	Insomnia		Depression			Suicide		Deaths, n		Confirmed cases, n	
	Translated keyword	Days with significant increases in searches, n		Translated keyword	Days with significant increases in searches, n	Translated keyword	Days with significant increases in searches, n	From March 20 to April 19	As of March 20	From March 20 to April 19	As of March 20
Australia	insomnia	5		depression	6	suicide	0	60	7	5819	791
Brazil	insônia	10		depressão	0	suicídio	0	2451	11	37,861	793
Canada	insomnia	4		depression	4	suicide	1	1552	12	34,676	943
France	insomnie	9		dépression	0	suicide	2	19,244	450	139,196	12,612
Germany	Schlaflosigkeit	4		Depression	0	Selbstmord	10	4519	67	125,336	19,848
Hong Kong	失眠	1		抑鬱	6	自殺	3	0	4	769	256
Iran	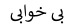	17		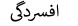	9	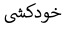	10	3685	1433	62,567	19,644
Italy	insonnia	11		depressione	1	suicidio	5	19,628	4032	131,951	47,021
Japan	眠れない	1		うつ病	0	自殺	0	203	33	9834	963
New Zealand	insomnia	2		depression	1	suicide	1	12	0	1392	39
Russia	бессонница	1		депрессия	0	самоубийство	0	360	1	42,600	253
Singapore	insomnia	3		depression	0	suicide	2	11	0	6203	385
South Korea	불면증	1		우울증	3	자살	2	140	94	2009	8652
Spain	insomnio	16		depresión	4	suicidio	0	19,410	1043	178,264	20,410
Taiwan	失眠	4		憂鬱	2	自殺	4	4	2	285	135
Thailand	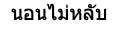	6		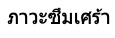	2	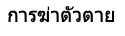	1	46	1	2443	322
Turkey	uykusuzluk	2		depresyon	0	intihar	0	2013	4	85,947	359
United Kingdom	insomnia	4		depression	0	suicide	0	18,298	194	116,084	3983
United States	insomnia	11		depression	4	suicide	1	40,596	349	739,536	19,273

**Figure 3:**
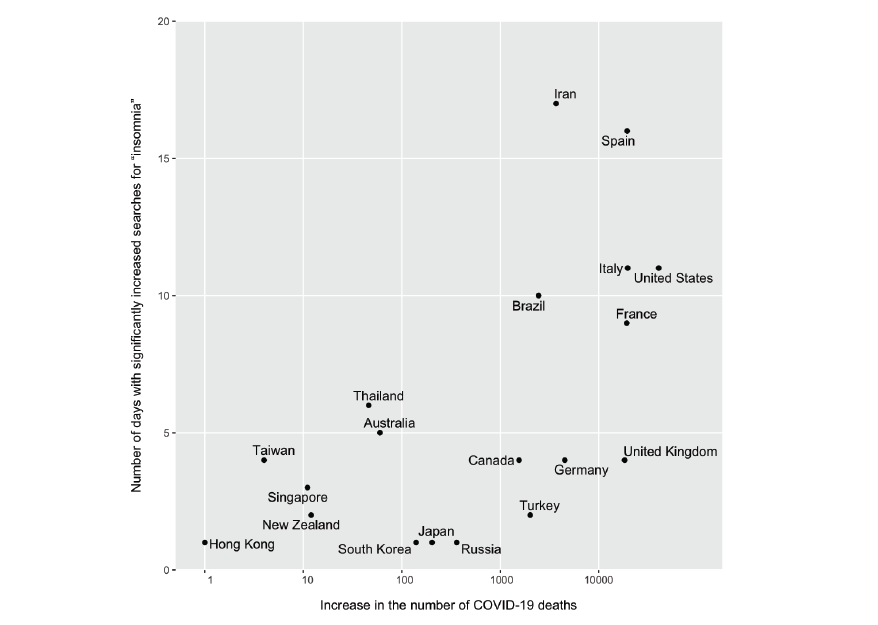
Positive correlation between increases in COVID-19–related deaths and the number of days with an increase in searches for insomnia in 19 countries (Spearman correlation coefficient=0.64, *P*=.003) from March 20 to April 19, 2020.

Figure 3 shows that increases in deaths were positively correlated to the number of days with increases in searches for insomnia in the 19 countries from March 20 to April 19 with a Spearman correlation coefficient (ρ) of 0.64 (*P*=.003). Similarly, the number of cumulative death cases as of March 20 was temporally positively correlated to increases in insomnia searches from March 20 to April 19 (ρ=0.60, *P*=.007).

By contrast, there was no significant correlation between the increased searches for depression and increases in deaths (ρ=–0.12, *P*=.63), nor a correlation between the increased searches for suicide and increases in deaths (ρ=–0.07, *P*=.79).

## Discussion

### Principal Findings

To the best of our knowledge, this is the first study to use a real-time collection of population-level data to investigate the impacts of the COVID-19 pandemic on mental health across 19 countries. The multinational and longitudinal design demonstrates with a high temporal resolution how the pandemic influences global mental health. Furthermore, this study compared the associations between the impact of the COVID-19 outbreak and its varied impact on mental health indicators, namely insomnia, depression, and suicide. The increase in Google searches for insomnia, rather than for depression and suicide, was significantly correlated to the number of deaths related to COVID-19. These findings indicate the extent of the pandemic’s impact on the general population’s mental health.

Insomnia is a common, preceding symptom or precipitating factor in new onset mental illness, such as depression, anxiety, and posttraumatic stress disorder [11], whereas suicide is the most severe outcome of psychiatric disorders over a lifetime [12]. Stress‐related sleep problems are common [13], and poor sleep quality has been identified as having mental health consequences as a result of social isolation [14]. In the absence of a vaccine and effective treatment for COVID-19, one of the most vital strategies for slowing the pandemic is social distancing. However, even for households free of the virus, the pandemic is likely to function as a major stressor, especially in terms of economic difficulties. Such effects may be exacerbated by self-isolation policies that can increase social isolation and relationship difficulties. Loneliness and social isolation may worsen the burden of stress and often exacerbate insomnia. Future research should explore search behaviors associated with terms such as fear, anxiety, and stress, which are highly relevant to COVID-19 and insomnia, to strengthen the qualitative correlation between insomnia and the COVID-19 pandemic.

Our results indicate that insomnia is a more sensitive indicator than depression or suicide of populations’ mental health during the COVID-19 pandemic. However, unlike the patterns of Google searches for “face mask” and “wash hands,” which reached all-time highs during this pandemic [10], searches patterns for insomnia, depression, and suicide usually consisted of multiple fluctuating components (eg, seasonality) and may increase in imperceptible manners. ARIMA models are capable of modeling both seasonal and nonseasonal data and can perform time-series forecasting to quantify whether imperceptible search queries are higher than expected [15,16].

The increased searches for insomnia and suicide demonstrated different patterns in this study (Figures 1 and 2). The surge in suicide-related searches in some countries (Italy, Germany, and Iran) may be attributed to media reports of suicides (ie, an Italian nurse on March 24, a German minister on March 29, and an Iranian student on April 6). These findings are consistent with previous studies that report on the correlation between suicide search trends and actual suicides [17], indicating that media reports of suicides can lead to spikes in suicides [18]. On the basis of these findings, media professionals should follow the WHO’s media guidelines [2] for preventing suicide.

### Limitations

There are several methodological limitations that should be noted when interpreting this study’s findings. First, we recognize that the Google Trends data do not represent a random sampling of the population and may exclude important vulnerable groups without access to the internet or those who were not actively engaged in searching. Second, we were unable to determine the sociodemographic characteristics of those conducting the searches. COVID-19 disproportionately affects poor and vulnerable populations; additionally, patients with serious mental illness may be among the hardest hit [19]. Our results did not include the entire population, or all internet users, of every country. Third, individual search queries for insomnia, depression, or suicide may not accurately reflect the actual mental health status of internet users. Factors other than the COVID-19 pandemic, including cultural differences that affect the expression and evaluation of symptoms, as well as more complicated bio-psycho-social factors may influence mental health and thus internet search behaviors. However, the collective phenomenon of internet search behavior still can be a meaningful surrogate for mental health across large populations.

### Conclusion

In conclusion, our analysis suggests that insomnia could be a part of routine screening for mental health during the COVID-19 pandemic. Monitoring Google Trends has the benefit of allowing for rapid longitudinal tracking at the international level during this unprecedented health crisis.

## Abbreviations

ARIMA: autoregressive integrated moving average
WHO: World Health Organization
